# Risk Factors for Suicidal Behaviours after Natural Disasters: A Systematic Review

**DOI:** 10.21315/mjms2020.27.3.3

**Published:** 2020-06-30

**Authors:** Hamid Jafari, Mohammad Heidari, Samaneh Heidari, Nasrin Sayfouri

**Affiliations:** 1 Sirjan School of Medical Sciences, Sirjan, Iran; 2 Community-Oriented Nursing Midwifery Research Center, Shahrekord University of Medical Sciences, Shahrekord, Iran; 3 Trauma and Injury Research Center, Iran University of Medical Sciences, Tehran, Iran; 4 Department of Foreign Languages, School of Health Management and Information Sciences, Iran University of Medical Sciences, Tehran, Iran

**Keywords:** natural disasters, suicide, mental health, PTSD

## Abstract

Natural disasters have multiple psychological effects including increased risk of suicide among victims. Reviews have shown that suicidal behaviours can be an aftermath of natural disasters. The present study attempted to identify the suicide-related risk factors after natural disasters. This study was a systematic review probing English language articles related to suicide and its risk factors after natural disasters and published between 1 January 1990 and 27 September 2018 in Google Scholar, PubMed, Web of Science, Science Direct, Scopus, ProQuest and Cumulative Index of Nursing and Allied Health Literature (CINAHL) databases. After reviewing and screening the collected studies by means of specific criteria, only 30 studies were qualified to enter the survey. It was found that most of these studies had investigated suicide after earthquake. Gender, age, serious mental disorders, depression, post-traumatic stress disorder (PTSD), loss of family members, low economic status, low social support, and injury to the person and the family/relatives were identified as the most important risk factors for suicide after natural disasters. Women, adolescents, elderly, people with depression and PTSD, those suffer from low social support and parentless people were found to be among the ones being highly vulnerable to suicide after natural disasters. There is, therefore, a need for providing psychosocial support for these people after such disasters.

## Introduction

Disasters have been one of the main concerns of public health in recent years as they have had substantial effects on the health of the affected people (1). Although the number of mortality has been slightly declining during the recent years, the frequency of the disasters, the number of the affected people and the resulting economic damages are increasing. Based on the reports from the Center for Research on the Epidemiology of Disasters (CRED) and United Nations International Strategy for Disaster Reduction (UNISDR) over the last two decades, natural disasters have killed more than 1.3 million people and have left over 4.4 billion people injured, homeless, and in need of urgent assistance (2–4).

The negative effects of disasters on human health have been attempted to be recognised (5, 6). Respiratory, digestive and cardiovascular symptoms were reported up to five years after disasters among the affected population (7, 8). However, the long-term effects of disasters, different from the physical ones, have not been dealt with adequately in the studies. The health effects of natural disasters are not limited to deaths and injuries. They also include psychological problems such as anxiety, depression, post-traumatic stress disorder (PTSD), aggression, insomnia, and suicide (9). More specifically, exposure to natural disasters may increase the risk of suicide among the survivors through thinking about suicide and, finally, suicide attempts (10). The extent to which how disasters cause problems related to suicide behaviour is still under debate.

As psychophysiological disorders and economic problems are well-known risk factors of suicide, one might expect that suicidal behaviour rates increase after natural disasters (11–14). Although temporary symptoms are more common, the psychological consequences of long-term reactions, which may result in committing suicide, can be more harmful (13). These responses may result from the stress directly related to the disasters (death or injury of family members, or loss of capital or occupation) or from disturbances caused in the structure of social life (15), or the stresses from the process of seeking help, housing, or repaying insurance costs (16).

It is, therefore essential to identify clearly the risk factors which may potentially lead to suicide attempts among the disaster survivors (17). In addition, it is essential to identify the relationship between suicide, PTSD and depression. The present systematic review study aimed to find a better understanding of post-disaster psychological vulnerability by identifying risk factors for suicidal after natural disasters. The findings of this study can provide information for post-disaster psychological interventions for vulnerable groups.

## Methods

### Study Design

The present review study was conducted based on the preferred reporting items for systematic reviews and meta-analysis (PRISMA) guidelines (18). We searched Google Scholar, PubMed, Web of Science, Science Direct, Scopus, ProQuest and Cumulative Index of Nursing and Allied Health Literature (CINAHL) databases between 1 January 1990 and 27 September 2018.

### Search Strategy and Inclusion/Exclusion Criteria

The search was conducted by the following keywords and their components: (Suicide or Suicide ideation or Suicide attempt or Suicide death or Self-destruction or Self-murder or Self-slaughter) and (Disasters or Natural disasters or Emergencies or Natural emergencies or Crisis or Mass casualty incidents or Trauma).

To obtain authoritative information, this review included only peer-reviewed journal articles in English language having studied suicide and suicide risk factors after natural disasters, while the articles with low quality and reliability (based on research team ideas or acquired scored less than 8 from 12 based on the PRISMA checklist), editorial articles, as well as the studies conducted before 1990 were excluded from the study. The questions in Table 1 were used to assess the selected studies.

### Study Selection Process

**Figure 1:**
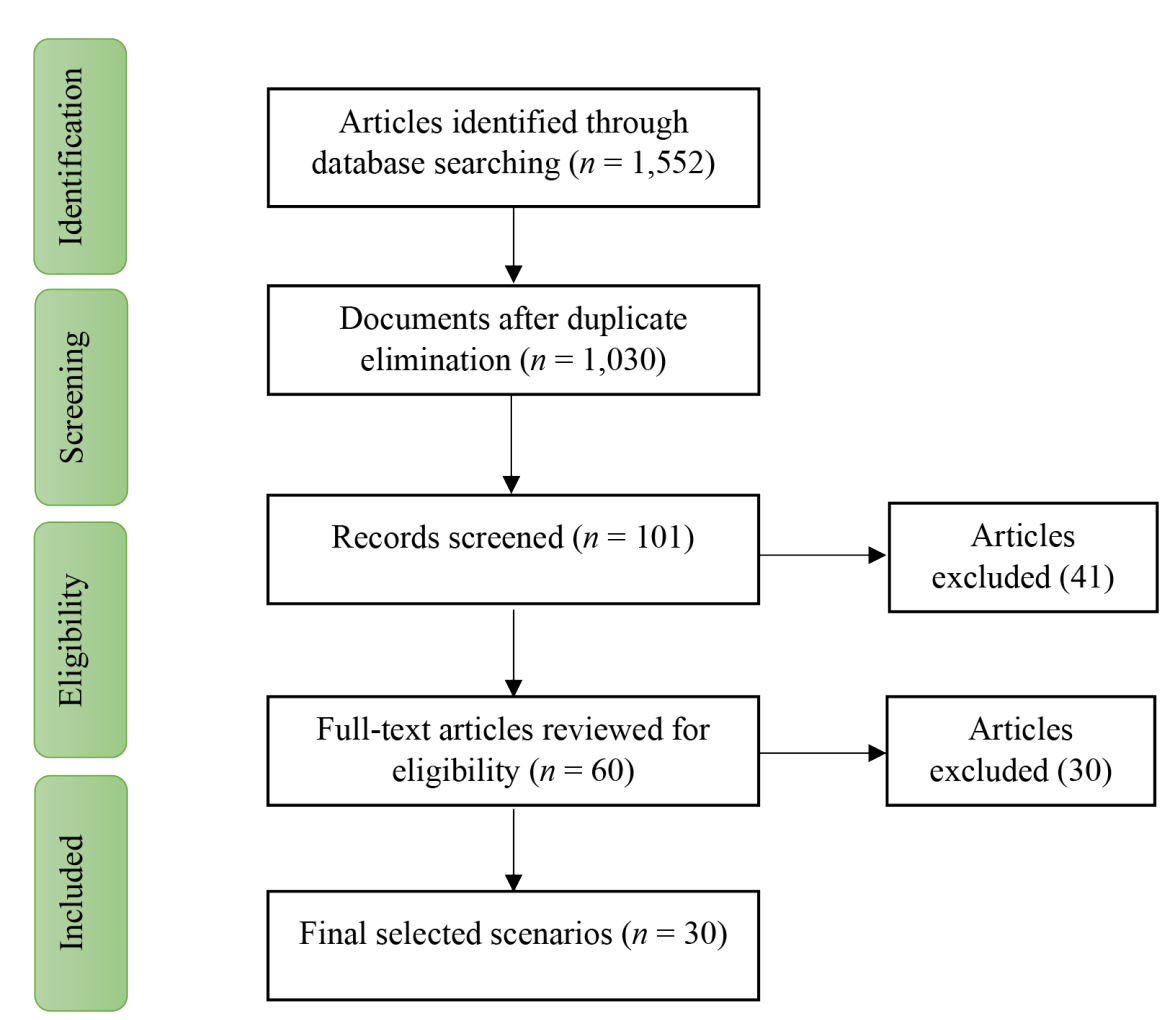
Process of article selection based on PRISMA guidelines

The initial search by using the proper combination of keywords among the original scientific websites led to the identification of 1,552 studies. Then, the articles with identical titles as well as the duplicate articles were deleted. At this phase, 522 studies were removed and finally, 1,030 articles were selected to be studied systematically. Then, the titles of these studies were evaluated in a systematic screening to identify the studies related to suicide after natural disasters. At the end of this phase, 101 articles were found to be relevant. The abstracts of these articles were examined. Sixty articles received the minimum score and were selected for full text reading. These articles were read independently by two authors of the present study. Among these articles, only 30 ones were found to deal with suicide after the natural disasters. Figure 1 illustrates the process of collecting and reviewing studies based on PRISMA instruction.

## Results

The results in Table 2 indicated that in 21 studies out of the 30 selected ones, suicide has occurred after earthquake. The remaining articles were shown to be involved with other hazards.

According to Table 3, Japan had the largest share in the selected studies, i.e., eight studies (12, 19–25) out of 30 followed by the United States showing to have the second largest share of the selected studies.

The results related to the identified factors can be seen in Table 4. The factors responsible for suicide attempts after the disasters were found to include: age, gender, PTSD, suffering from serious psychiatric disorders before the disaster, depression, damages to the homes or the properties, injuries occurring to the person/the family/the relatives, race, economic status, pre-disaster physical incapacity, unemployment, loss of family members, lack of post-disaster recovery services, religion, low social support, low education and the number of the victims. Gender (female in most studies) was introduced as the most frequent factor in 14 articles (10, 14, 21, 22, 24–33). The next major risk factor was found to be ‘the incidence of serious pre-disaster mental disorders’ being mentioned in 10 studies (10, 14, 19, 21, 31, 33–37).

The other common risk factors included age (10, 22, 23, 26, 27, 31, 38, 39), low social support (17, 21, 26–28, 33, 34, 36) and injury to the person, his family or the relatives (14, 21, 30, 31, 37, 40, 41), mentioned in nine, eight and seven studies, respectively. Depression was studied in six articles (10, 26–28, 30, 33), PTSD in six articles (26–28, 31, 32, 42), economic status (20, 21, 26, 39), and loss of family and relatives, each in four articles (28, 30, 31, 34), damages to the homes and the properties (30, 37, 38), and lack of recovery services, each in three articles (25, 26, 39), unemployment, (19, 39) low education (26, 31), and the number of victims in two articles each (22, 30), and, finally, race (29) and religion (26) each in one study. The range of the periods of the occurrence of the above-mentioned factors in these studies varied from one month to 29 years after the occurrence of the natural disasters. Most studies evaluated the factors between one to three years after the disaster.

Based on the information found in the selected articles in Table 4 it was realised that the majority of them pointed to an increase in suicide after natural disasters. Very few studies, such as those conducted after the Hanshin earthquake in Japan reached the conclusion that suicide was reduced after natural disasters (28). However, it was recognised that those studies which referred to an increase in suicide after natural disaster did not mean that the increase has happened immediately after the disaster. Instead, in a period after a disaster, i.e. between one month and two years, suicide shows a considerable decrease. But after that period, suicide starts to escalate.

## Discussion

The results of the study indicate that most of our selected articles have focused on suicide prevalence after the occurrence of earthquake and few of them considered other natural disasters such as hurricanes, cyclones, floods and tsunamis. The emphasis of studies on earthquake is probably due to the high number of earthquake victims, its disastrous long-term consequences and its high incidence in the societies. However, no study, to the best of our knowledge, has been conducted to explore the slow and long-term impacts of natural disasters such as those of drought despite its frequent occurrence and high suicide rates in some societies (43, 44). On the other hand, it has been stated in literature that long-term crises, such as droughts, can increase the incidence of depression and other psychiatric disorders (45). Such factors merit to be explored as the risk factors of suicide.

The findings, moreover, designate a significant geographical distribution regarding suicide rates after disasters. Among 30 studies, 19 ones were conducted in the East Asian region (Table 4). These countries are generally among the countries with high suicide rates in the world such as Japan and China (46–48). The United States, however, is not among the high-risk countries for suicidal rate. Therefore, the reason for the considerable number of investigations on suicide after disasters in this country (Table 4) can be due to the fact that the United States is one of the countries with high level of natural disasters (48, 49).

It should be noted that China, Taiwan, Japan and the United States are all among the countries which are highly prone to natural disasters (43). Nevertheless, The effect of natural disasters on suicide rate in European countries which are among high risk countries of suicide and Middle Eastern countries, which are among the low-risk countries of suicide, is still unknown and should be scrutinised to provide a comprehensive paradigm about the effect of natural disasters on suicide in different countries and cultures.

Based on the results of this review study, the female gender was found to be the factor having the greatest effect on suicide rate after natural disasters. Based on the relevant selected studies, this can be due to more psychological vulnerability of women to natural disasters, increased violence against women, increased poverty among women, increased rape rates, loss of the householders, as well as women’s less access to health services, adequate nutrition and safe shelters (49–51). Contrary to this finding, some studies reported that the rate of suicide after natural disasters among women were less than that of men. This can justify the higher resilience of women compared to men (12). Even, in a few studies, male gender, aged between 18 and 39, was identified as a risk factor. The main cause of suicide at this age in post-disaster situations may be related to the inability of these people, as the householders, in providing family expenses and restoring the damaged or lost assets caused by the disasters. Obviously, the risk of depression increases with the destruction of homes and assets, loss of family and relatives, and the occurrence of poverty after disasters.

Having a serious mental disorder before a disaster as the second most common predictor of suicide after a disaster was mentioned in ten studies. Such mental disorders, having been evaluated in previous studies on suicidal risk factors, include anxiety, depression, personality disorders, sexual problems, alcohol dependence and addiction (52). In the present study, depression was considered as a separate factor being mentioned in six studies. Even one study identified depression as the strongest predictor of suicide after natural disasters (24). Other studies identified depression as a risk factor for suicidal ideation in a person. Depression can increase the risk of suicide by increasing fatigue, lowering the quality of life, increasing the person’s desire to consume drugs and alcohol and increasing the incidence of multiple physical disorders such as digestive problems (53–57).

Age is another risk factor being emphasised in nine studies. They considered adolescence and aging periods as the vulnerable ages against suicide after natural disasters. The risk of depression and suicide rises in the elderly due to less ability to fulfill daily activities and less adaptive capacity as well as the need for more social support and dependence on others after disasters (58, 59). In addition, the elderly are at the increased risk of suicide because of various physical and mental disorders (60, 61). In adolescents, the factors such as loss of family and social support, imprisonment, poor life skills, family history of suicide, the Internet and mass media, detected psychiatric disorders, disastrous life events, history of child abuse, academic stress and drug abuse were identified as the risk factors of suicide (62, 63).

Low social support was identified as a strong predictor of increased suicide after natural disasters. After natural disasters, the social support for the victims of disasters decreases due to loss of family members and friends as well as the reduction of social interactions, which can lead to depression and an increased risk of suicide (64).

The next risk factor was found to be injury to the person, the family members or the relatives which can make a person more vulnerable than before. In such cases, especially when there is a resource constraint, the person’s suicidal tendency increases and he needs additional social protection especially in more defenseless families (65).

PTSD was studied more than other psychological problems and is probably the most common disabling psychological disorder occurring after natural disasters (65, 66). Six of the selected studies demonstrated a strong correlation between PTSD and suicide after natural disasters. Risk factors for PTSD after natural disasters was stated to include psychological factors such as neuroticism, guilt, concentration problems, coping strategies, obsessive-compulsive and psychiatric comorbidity. It was shown that women are more likely to suffer from PTSD after natural disasters compared to men. On the other hand, low social support was also found to be associated with a high probability of PTSD. Furthermore, higher level of exposure to a disaster is consistently associated with the risk of developing PTSD (67).

The economic status of the victims was another risk factor for suicide after natural disasters. It has been mentioned that poverty can be a factor in suicidal behaviour depending on the cultural conditions of society but poverty typically tends to be more intense after natural disasters which may not be recovered without external assistance (68, 69). Ongoing surveillance is, therefore, needed, not only for the sufferers who are directly affected, but also for those who may be affected by the economic aspects of the disaster (70).

Damage to the properties and assets as well as unemployment related to the economic conditions of the victims are other factors associated with the occurrence of suicide. However, other risk factors such as lower education, race, and religion should be highlighted in future studies.

## Conclusion

To sum up, the most significant risk factors for increasing the risk of suicide after natural disasters were found to be gender, age, serious mental disorders, low economic status, depression, PTSD, loss of family members, low social support and injury to a person, family or relatives.

It is, therefore, essential that psychological health and suicidal behaviours be assessed for several years after the disasters. Psychosocial interventions for the victims can reduce mental disorders, increase their ability to adapt to the created conditions, improve mental health and increase their resilience to disasters. In other words, addressing the psychological consequences of survivors of natural disasters is indispensable for normalising the reactions and preventing later complications which, may otherwise, lead to decrease in the quality of life and the efficiency of individuals. Last but not least, some affected groups who suffer more and are more vulnerable to disasters (such as women, adolescents, elderly, people with depression and PTSD, victims, and parentless individuals) should be highly protected.

## Figures and Tables

**Table 1:** Questions used to assess the selected articles (22)

No	Question	Score	
		Yes = 1	No = 0
1	Are the research questions clearly stated?		
2	Is the approach appropriate for the research question?		
3	Is the study context clearly described?		
4	Is the role of the researcher clearly described?		
5	Is the sampling method clearly described?		
6	Is the sampling strategy appropriate for the research question?		
7	Is the method of data collection clearly described?		
8	Is the data collection method appropriate to the research question?		
9	Is the method of analysis clearly described?		
10	Are the main characteristics of the population well described?		
11	Is the analysis appropriate for the research question?		
12	Are the claims made supported by sufficient evidence?		

**Table 2:** The disaster types and the number of the related articles

Type of hazard	Number of studies
Earthquake	21 studies
All natural hazards	1 study
Tsunami	3 studies
Flood	2 studies
Hurricane	5 studies
Super cyclone	2 studies

**Table 3:** Countries in which suicide was investigated after the disasters with their shares in the selection

Country	Number of studies
Taiwan	4
Japan	8
China	4
Chili	1
USA	6
Turkey	2
Sri Lanka	1
India	2
Nicaragua	1
Italy	1

**Table 4:** Summary of data extraction of the selected articles (1990–2018)

Author (year)	Setting	Method	Time spent (after the disaster)	Identified risk factors for suicide	Findings
Chen et al. (19)	Taiwan	Time series	9 years	Age	Earthquake resulted in a significant decrease in standardised suicide mortality ratios. Gender was not a risk factor for suicide.
Ohto et al. (20)	Japan	Time series	3 years	Unemployment, having a serious psychological illness	Suicide prevalence decreased during the first 2 years and then increased.
Tang et al. (21)	China	Empirical study	3 years	Age, gender, depression, PTSD	Suicidality is independently associated with emotional abuse, neglect, depression and PTSD symptoms in this disaster-exposed young population. Physical neglect or abuse were not related to suicidality.
Fujiwara et al. (23)	Japan	Follow-up study	3 years	Gender, having a serious psychological illness, injuries of individual or their relatives	Trauma experience before the earthquake, behaviour problems at baseline, subjective socioeconomic status, maternal mental health, and parenting practice at baseline were not associated with suicide risk in the crude model.
Guo et al. (23)	China	Cross- sectional	8 years	PTSD, depression, gender, perceived non-recovery, low social support, low education	Targeted, long-term suicide prevention programmes for adult survivors should be further developed.
Ran et al. (24)	China	Follow-up study	6, 12 and 18 months	PTSD, depression, gender, low social support, loss of relatives	Depression symptoms were the strongest predictor of suicidal ideation after earthquake. An increased rate of suicidal ideation after the earthquake may be mainly due to depression but not to PTSD symptoms. There was no significant differences of rates of suicidal ideation between adolescents who lost their family members and those who did not lose their family members. Evidence indicates that earthquakes, by themselves, do not cause suicides.
Matsubayashi et al. (25)	Japan	Panel data	29 years	Age, gender, number of disaster victims	Areas affected by natural disasters experienced a temporary increase in suicide rates in the same year, but the rates decreased over the next two years. Subsequently, however, the suicide rates in the areas increased again in the third and fourth year after the disaster. Five years after the disaster, the overall suicide rate was not associated with the size of the damages.
Chou et al. (12)	Taiwan	Cohort study	2–15 months	Age, gender, pre- disaster physical disability status, having a serious psychological illness	The risk of suicide increased with age. Women had a 50% lower risk of suicide than men.
Aoki et al. (26)	Japan	Time series	1 year	Economic conditions	The risk of non-fatal suicide attempts using high mortality methods was significantly higher for four months, by three to four times after a series of disasters, and then decreased. The total number of death by suicide reported by the police decreased in the first year after the disasters.
Brown et al. (27)	Chile	Follow-up study	1 year	PTSD	Groups with pre-disaster PTSD should be prioritised for receipt of mental health resources following a natural disaster. Pre-disaster panic disorder is not associated with elevated thoughts of death or suicide following a natural disaster.
Nishio et al. (28)	Japan	Time series	5 years	Age	Suicide rates in Kobe significantly decreased in the two years after the earthquake. An influence on suicide rate after the disaster clearly appeared in the middle-aged men.
Yang et al. (14)	Taiwan	Time series	2 years	Number of disaster victims	Higher suicide rates were observed over a 10-month period following the disaster.
Krug et al. (10)	USA	Time series	4 years	Age, gender, depression, having a serious psychological illness	Suicide rates increased in the four years after floods in the two years after hurricanes and in the first year after earthquakes. The suicide rates did not change significantly after tornadoes or severe storms. Strong social support is a protective factor against suicide.
Shoaf et al. (29)	USA	Time series	3 years	Gender, ethnicity	The rates of suicide are lower in the three years following the earthquake than those in the three years prior to the earthquake.
Vehid et al. (30)	Turkey	Cross- sectional	2 months	Gender, depression, damage to home or property, loss of relatives, injuries of individual or their relatives	-
Suzuki et al. (31)	Japan	Cross- sectional	3 years	Gender	-
Hyodo et al. (32)	Japan	Time series	3 years	Gender	Suicide rate decreased in men after earthquake.
Akbiyik et al. (33)	Turkey	Cross- sectional	3 years	Loss of relatives, having a serious psychological illness, low social support	Temporary symptoms are more common than long-term reactions. Earthquake experience was not a risk factor in suicidal ideation.
Castellanos et al. (34)	USA	Time series	16 months	-	Suicide rates did not increase after hurricane.
Chen et al. (54)	Taiwan	Time series	First month	Injuries of individual or their relatives, damage to home or property	It showed that there were no significant associations between occurrence of suicidal ideation and gender, age, and obsessional personality.
Rodrigo et al. (36)	Sri Lanka	Time series	1 year	-	No significant differences were found between the number of suicides before and following the disaster or between areas affected and unaffected by the tsunami.
Kar (59)	India	Qualitative longitudinal observational study	3 years	-	Suicidal ideation, plans, and attempts are common after cyclone. The status of post-disaster morbidity and higher vulnerability of these communities suggested the need to improve disaster. Preparedness and management along with focused attention to psychosocial sequelae.
Lau et al. (38)	China	Cross- sectional	1 month	Gender, low education, PTSD, having a serious psychological illness, loss of relatives	Protective factors included perceived social support, frequent exposure to news contents that are touching, and perceived sense of security obtained from teachers.
Kessler et al. (39)	USA	Follow up study	1 year	Age, economic conditions	The prevalence of suicidality, finally, was significantly higher in the follow-up than baseline survey both with regard to suicidal ideation. Suicidality was found to be unrelated to sex, race/ethnicity, education, and health insurance status.
Gordon et al. (17)	USA	Cross- sectional	-	Low social support	Communities pulling together during a natural disaster can reduce interpersonal risk factors associated with the desire for suicide.
Caldera et al. (40)	Nicaragua	Time series	1 year	Having a serious psychological illness	-
Shioiri et al. (41)	Japan	Time series	2 years	-	Suicide decreased among survivors especially for men.
Stratta et al. (42)	Italy	Cross- sectional	1 year	PTSD, gender	Religious involvement was found to be related to better coping with stress and less depression, suicide, anxiety, and substance abuse.
Kar (59)	India	Cross- sectional	1 year	Having a serious psychological illness, low social support	Awareness of increased suicidality, attention to associated risk factors, and support may help in the prevention of suicide following disasters.
Warheit et al. (57)	USA	Time series	-	Having a serious psychological illness, gender, depression, low social support	-
